# TAK1/AP-1-Targeted Anti-Inflammatory Effects of *Barringtonia augusta* Methanol Extract

**DOI:** 10.3390/molecules26103053

**Published:** 2021-05-20

**Authors:** Anh Thu Ha, Mi-Yeon Kim, Jae Youl Cho

**Affiliations:** 1 Department of Integrative Biotechnology, and Biomedical Institute for Convergence at SKKU (BICS), Sungkyunkwan University, Suwon 16419, Korea; anhthu95.vn@gmail.com; 2 School of Systems Biomedical Science, Soongsil University, Seoul 06978, Korea

**Keywords:** *Barringtonia augusta*, anti-inflammatory effect, TAK1

## Abstract

*Barringtonia augusta* methanol extract (Ba-ME) is a folk medicine found in the wetlands of Thailand that acts through an anti-inflammatory mechanism that is not understood fully. Here, we examine how the methanol extract of *Barringtonia augusta* (*B. augusta)* can suppress the activator protein 1 (AP-1) signaling pathway and study the activities of Ba-ME in the lipopolysaccharide (LPS)-treated RAW264.7 macrophage cell line and an LPS-induced peritonitis mouse model. Non-toxic concentrations of Ba-ME downregulated the mRNA expression of cytokines, such as cyclooxygenase and chemokine ligand 12, in LPS-stimulated RAW264.7 cells. Transfection experiments with the AP-1-Luc construct, HEK293T cells, and luciferase assays were used to assess whether Ba-ME suppressed the AP-1 functional activation. A Western blot assay confirmed that C-Jun N-terminal kinase is a direct pharmacological target of Ba-ME action. The anti-inflammatory effect of Ba-ME, which functions by β-activated kinase 1 (TAK1) inhibition, was confirmed by using an overexpression strategy and a cellular thermal shift assay. In vivo experiments in a mouse model of LPS-induced peritonitis showed the anti-inflammatory effect of Ba-ME on LPS-stimulated macrophages and acute inflammatory mouse models. We conclude that Ba-ME is a promising anti-inflammatory drug targeting TAK1 in the AP-1 pathway.

## 1. Introduction

Inflammation, which plays an important role in protecting the body from harmful external influences, is associated with pain, swelling, heat, redness, and various functional impairments, and presents acute and chronic responses [1,2,3]. Without effective treatment, acute inflammation can become chronic. Hyperactive and prolonged inflammatory responses are considered important factors in various diseases, such as autoimmune disorder, cancer, diabetes, arthritis, and several vascular diseases [4,5]. Innate and adaptive immunity are the two parts of the immune system. The innate immune mechanism, which controls the activities of inflammatory response cells, comprises macrophages, neutrophils, and dendritic cells [6].

Toll-like receptors (TLRs) are proteins that have vital roles in the innate immune system. [6]. Lipopolysaccharide (LPS) is a major part of the TLR4 ligand [7]. Mitogen-activated protein kinase (MAPK) signaling was activated in the course of LPS-induced inflammation, because LPS binds to TLR4 and stimulates the recruitment of both TRIF adaptor proteins and cytoplasmic MyD88 [8]. MAPK families include extracellular signal-regulated kinase (ERK), c-Jun N-terminal kinase (JNK), and p38 kinase [9]. When TAK1 is activated, a sequential signaling cascade composed of mitogen-activated protein kinase kinases (MAPKKs) and kinase IKK is activated [10]. MAPKKs or IKK phosphorylate MAPK (JNK, ERK, and p38 or inhibitor of κBα IκBα) to activate activator protein 1 (AP-1) [11]. Activation of AP-1 increases when the MAPK signaling pathway is activated. The AP-1 signaling pathway consists of ATF, c-Fos, c-Jun, and JDP families [12,13]. Because many inflammatory diseases in humans occur with the activation of AP-1 [14], targeting the MAPK/AP-1 pathway is a promising and attractive therapeutic anti-inflammatory method.

The onset and intensification of inflammation in the body occasionally activate macrophages and release more cytokines, such as tumor necrosis factor-alpha (TNF-α), interleukin 6 (IL-6), IL-1beta, IL-12, and interferons [15]. Inflammatory genes include cyclooxygenase-2 (COX-2) and inducible nitric oxide synthase (iNOS) [16]. Cytokines and inflammatory genes are upregulated by activation of AP-1 transcription factors and nuclear factor kappa B (NF-κB). Therefore, decreasing inflammation is an important therapeutic goal and possibly prevents infection in the human body.

Traditional medicine and natural extracts offer benefits to healthcare and the treatment of various diseases [17]. Nowadays, studies have been developing with original plant extracts that are rich in antioxidants of a phenolic nature to study anti-inflammatory, antimicrobial, anticholinesterase effects, etc. Traditional medicines—more specifically, herbal extracts—have proven their effectiveness in the treatment of various diseases and are now gaining more attention among the scientific community for their potential in the development of medicine for treating urgent and contemporary diseases of the modern era. Most notably, Sytar et al. demonstrated the possible role of plant-derived natural antiviral compounds for the development of plant-based drugs against the representative coronaviruses group—specifically, COVID-19, which has caused a global pandemic that is currently ongoing [18]. Thymus species, which are culinary herbs and flavoring agents in Europe, North Africa, and Asia, have also proved to be promising therapeutic agents for neurodegenerative disorders (e.g., Alzheimer’s disease, which is currently ranked as the sixth leading cause of death in the United States) [19]. The anti-oxidative and anti-inflammatory effects of the variety of plants collected from 14 original research papers have been comprehensively reviewed and summarized by Allegra, providing an overview of the original plant extract’s antioxidants, which are phenolic in nature, for investigation of its anti-inflammatory, antimicrobial, anticholinesterase effects, etc. [20]. These previous works inspired us to attempt to utilize traditional medicine and natural extracts for anti-inflammatory applications. Interestingly, *Barringtonia racemosa* (a traditional plant in Malaysian villages) has been employed for human breast cancer treatment, drug discovery, and development [21]. In the same manner, in this research, we explored *Barringtonia augusta*, which is found in the wetlands of Thailand. Extracts of *B. augusta* exhibit antioxidant properties [22], although the molecular mechanisms by which they inhibit inflammatory responses through the AP-1 signaling pathway are not understood. In this study, we explored the anti-inflammatory effect of *B. augusta* methanol extract (Ba-ME). We investigated the roles of this compound in the regulation of the AP-1 signaling pathway in an LPS-treated macrophage RAW264.7 cell line and an LPS-induced peritonitis mouse model.

## 2. Results

### 2.1. Effect of Ba-ME on Cell Viability and Expression Levels of Inflammatory Genes in LPS-Treated Cells

**Figure 1:**
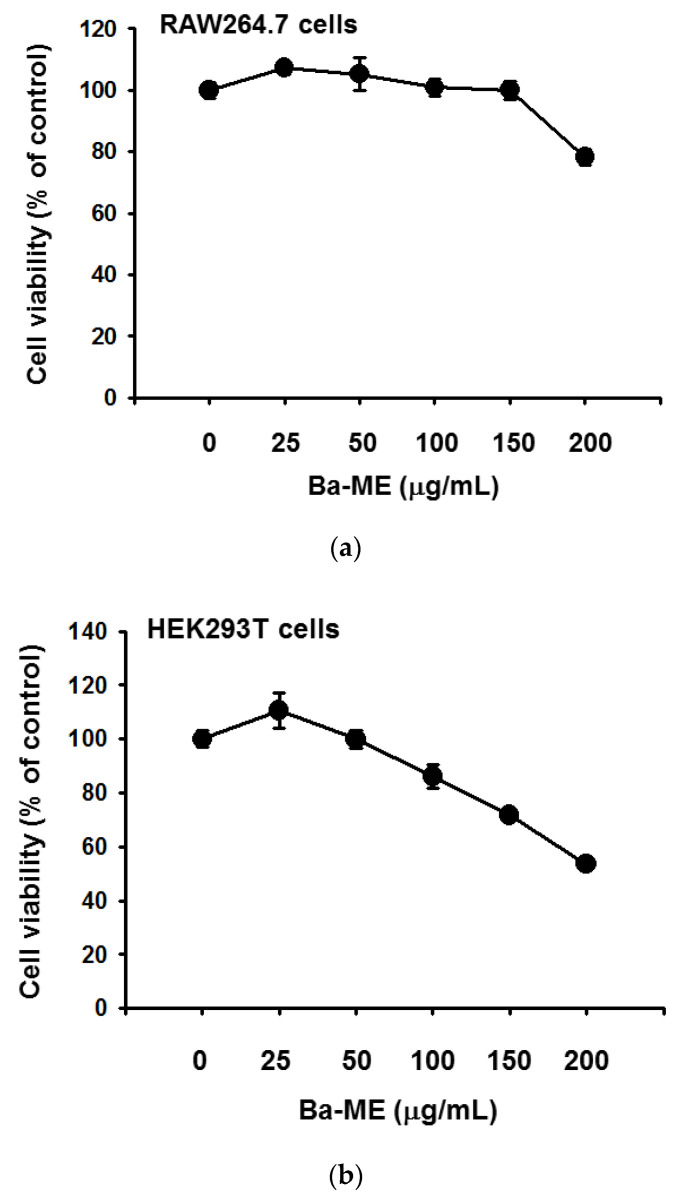

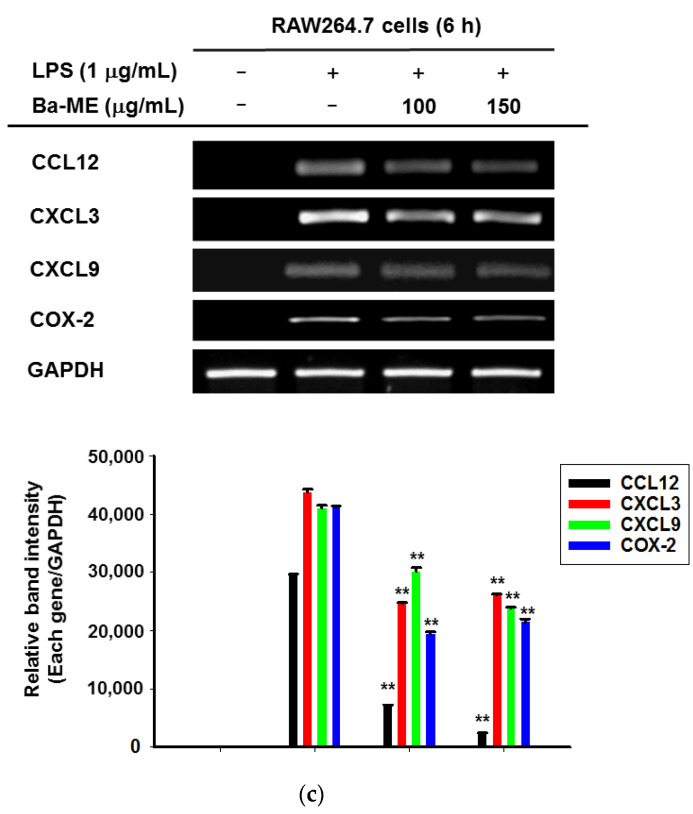
Effects of Ba-ME on cell viability and inflammatory gene expression in LPS-treated cells. (**a**) Treated RAW264.7 cells with Ba-ME with the indicated concentrations for 24 h. Used MTT assay to measure the cell viability. The values are presented as mean ± SD of 3 replicates. (**b**) HEK293T cells were treated with different Ba-ME concentrations for 24 h, and the MTT assay was performed to determine cell viability. The values are presented as mean ± SD of 3 replicates. (**c**) Employing RT-PCR to detect the mRNA expression levels of CCL12, CXCL3, CXCL9, COX-2, and GAPDH in LPS-stimulated RAW264.7 cells treated with Ba-ME (0–50 μg/mL). Band intensity (the bottom panel of (**c**)) was measured and quantified using ImageJ. ** *p* < 0.01 compared with control cells.

We inspected the cell cytotoxicity of Ba-ME (25–50 μg/mL) in RAW264.7 and HEK293T cells by MTT assay (Figure 1a,b). The viability of RAW264.7 and HEK293T cells was not affected notably by Ba-ME treatment compared with untreated cells.

We employed a reverse transcription PCR (RT-PCR) assay to examine the transcriptional level of pro-inflammatory genes. We measured the expression of the pro-inflammatory cytokines, such as chemokine (C-C motif) ligand 12 (CCL12), C-X-C motif chemokine ligand 3 (CXCL3), Chemokine (C-X-C motif) ligand 9 (CXCL9), Cyclooxygenase-2 (COX-2), and glyceraldehyde 3-phosphate dehydrogenase (GAPDH). CCL12 and COX-2 expression were decreased by Ba-ME treatment in a dose-dependent manner (Figure 1c). Because the mRNA expression levels of these cytokines are interconnected through the AP-1 pathway, we confirmed that Ba-ME attenuated the AP-1 pathway.

### 2.2. Effect of Ba-ME on Transcriptional Activation of AP-1

**Figure 2:**
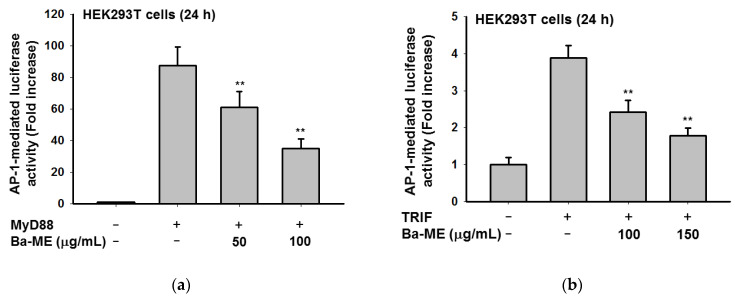
Effect of Ba-ME on activation of AP-1 pathways. (**a**,**b**) HEK293T cells were transfected with AP-1-Luc and β-gal constructs with Tag2-MyD88 (**a**) or CFP-TRIF (**b**). HEK293T cells were additionally treated with Ba-ME (0–100 µg/mL) for 24 h. AP-1-driven luciferase activity was measured by a luminometer. ** *p* < 0.01 compared with control cells. The values are presented as mean ± SD of 3 replicates.

Due to the regulatory role of the AP-1 transcription factor in inflammatory gene expression, we decided to inspect the suppressive effect of Ba-ME on such activation. In order to determine whether Ba-ME suppressed the activation of AP-1, a transfection experiment with the AP-1-Luc construction and HEK293T cells was conducted. The result showed that AP-1-mediated luciferase activity was intensified by co-transfection with TRIF and MyD88. In contrast, Ba-ME treatment inhibited this upregulation significantly (*p* < 0.01) with dose dependence (Figure 2). These results indicate that AP-1 activation is a vital pharmacological target of Ba-ME.

### 2.3. Regulatory Mechanism of Ba-ME in AP-1 Pathways

**Figure 3:**
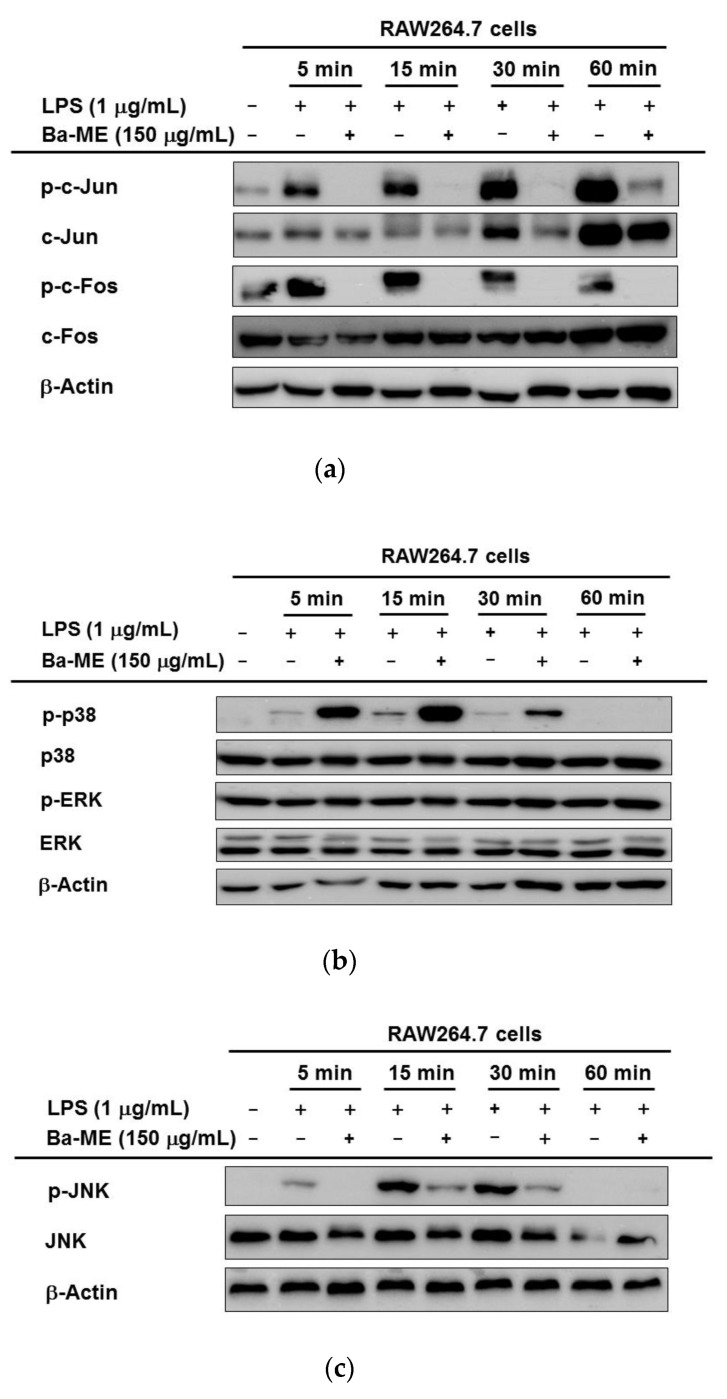
Effect of Ba-ME on intracellular signaling molecules’ activation in the AP-1 pathway. (**a**–**c**) Treated RAW264.7 cells with LPS (1 µg/mL) for 5, 15, 30, 60 min in the absence or presence of Ba-ME (150 µg/mL). A Western blot analysis determined the levels of phosphorylated forms and total forms of c-Jun and c-Fos (**a**). Using an immunoblot analysis of cell lysates to determine the phosphoprotein levels of p-38, ERK, JNK, and β-actin (**b**,**c**).

Whether Ba-ME can suppress the activation and translocation of AP-1 was our next investigation in this study. Figure 3a shows the increase in nuclear levels of the AP-1, c-Fos, and c-Jun subunits due to time-dependent (5, 15, 30, and 60 min) inhibition by Ba-ME. Similar time-dependent (5 min) inhibitory c-Fos and c-Jun expression patterns were confirmed from RAW264.7 cells by whole lysate extraction.

It is an important and non-trivial task to establish which intracellular molecules are targeted by Ba-ME in the AP-1 signaling pathway. We measured the levels of phosphorylated MAPKs (p-38, ERK, and JNK). We noticed that LPS obviously raised the phosphorylation of ERK, JNK, and p-38. In contrast, the phosphorylation of JNK was suppressed by Ba-ME strongly and time-dependently (5, 15, 30, 60 min), but that of p-38 and ERK (Figure 3b,c) in RAW264.7 cells was not after the treatment of Ba-ME. As phosphorylation of MAPKs is crucial in regulating the LPS-induced inflammatory mediators, our results confirmed that Ba-ME blocks the AP-1 pathway through the expression level of JNK.

**Figure 4:**
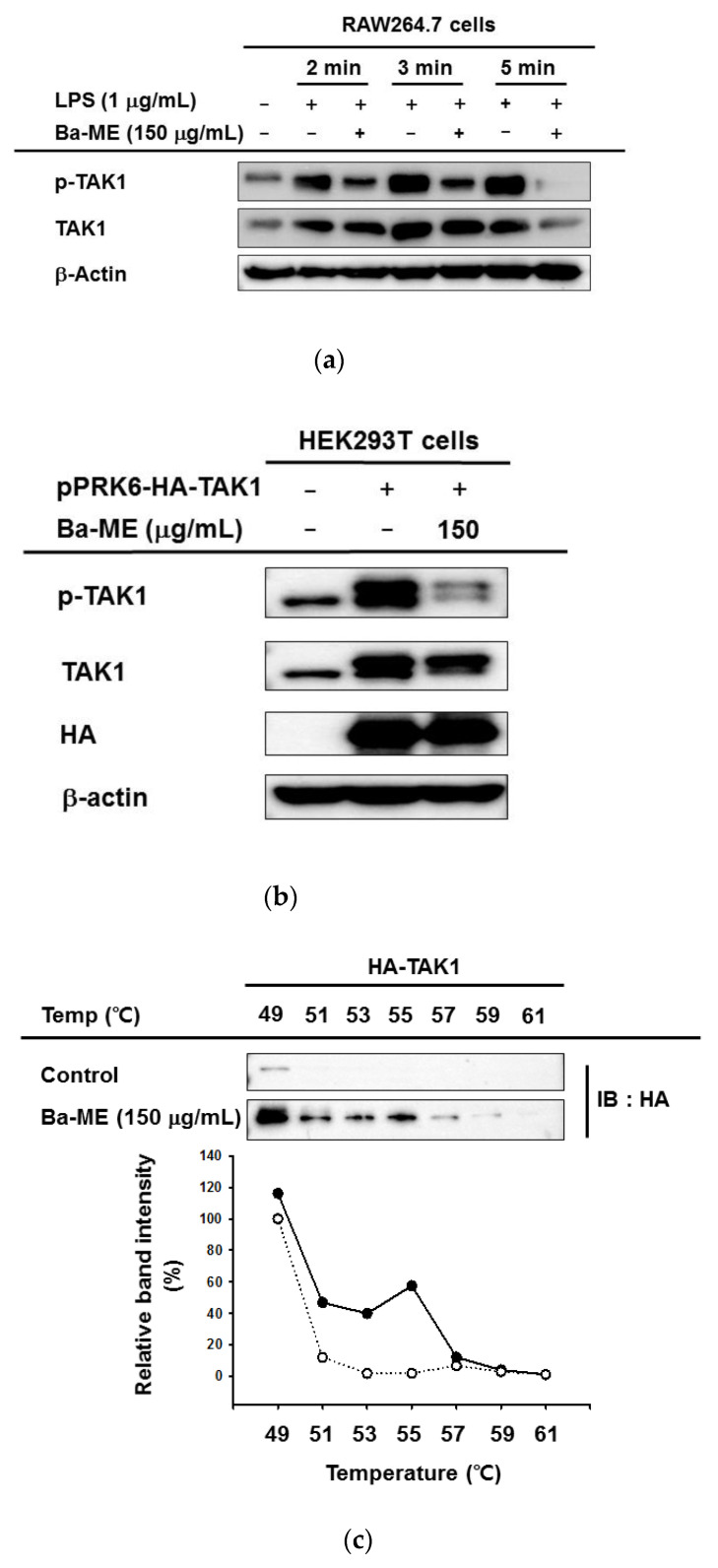
(**a**) RAW264.7 cells were incubated with Ba-ME in the absence or presence of LPS (1 µg/mL) for the designated times. After whole-cell lysates, employing immunoblotting to identify levels of total and phosphoforms of upstream TAK1 signaling enzymes. (**b**) After 24 h transfected pPRK6-HA-TAK1 constructs with HEK293T cells, treatmentt with Ba-ME was advisable, using a Western blot analysis to determine the levels of phosphorylated and total forms of TAK1-HA. (**c**) After overexpressing TAK1 in HEK293T cells, a CETSA was performed with Ba-ME (150 µg/mL), and dimethyl sulfoxide was used as a control. A Western blot analysis was conducted to examine the stabilization of Ba-ME on TAK1. Solid circles are Ba-ME group and hollow circles are control group.

We analyzed the phosphorylated forms of AP-1-related proteins to identify the targeted protein of Ba-ME in inhibiting the AP-1 pathway. As shown in Figure 4a, phosphorylated TAK1 was detected by LPS induction for 2, 3, and 5 min. TAK1 is the most upstream protein in the AP-1 pathway; it orders AP-1 signaling to progress, and its activation is required to activate macrophages. Using a Western blot assay, we observed that TAK1, the phosphorylated forms of AP-1 pathway-related proteins [11], were decreased by Ba-ME at 2, 3, and 5 min. This result proved that Ba-ME specifically targets TAK1. Moreover, the inhibition of TAK1 kinase alleviates the activation of downstream proteins.

### 2.4. Anti-Inflammatory Effects of Ba-ME by Targeting TAK1

The whole cell lysate immunoblotting, which used HEK293T cells to overexpress TAK1, was conducted to examine Ba-ME’s capability in inhibiting autophosphorylation of target enzymes. We overexpressed the plasmids expressing pPRK6-HA-TAK1 in HEK293T cells after 24 h and exposed the cell that was treated to Ba-ME (150 µg/mL) for another 24 h. The p-TAK1 level was reduced by the Ba-ME treatment (Figure 4b).

To assess the interaction of Ba-ME with TAK1 in intact cells, we performed a cellular thermal shift assay (CETSA) at 49 °C, 51 °C, 53 °C, 55 °C, 57 °C, 59 °C, and 61 °C. Figure 4c shows that Ba-ME treatment shifted the thermal stability of the target protein TAK1.

### 2.5. Ba-ME Alleviates Clinical Signs of LPS-Induced Peritonitis in a Mouse Model

**Figure 5:**
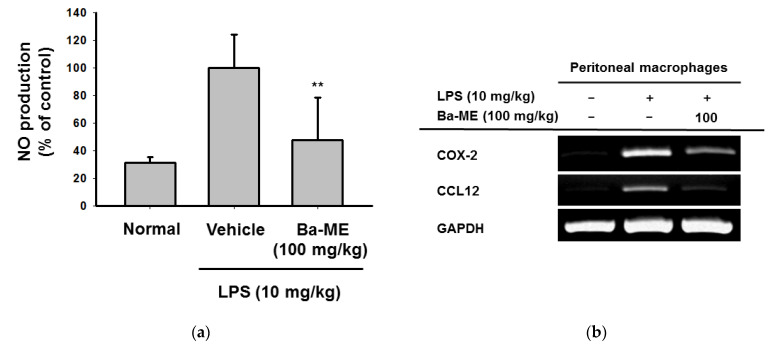
Effect of Ba-ME on regulating inflammation in an LPS-induced peritonitis mouse model. (**a**) Peritoneal macrophage inflammatory lesions were examined with an NO assay. (**b**) The mRNA expression levels of COX-2, CCL12, and GAPDH in peritoneal macrophages treated with Ba-ME (100 mg/kg) were determined using semiquantitative RT-PCR. ** *p* < 0.01 compared with control cells. The values (**a**) are presented as mean ± SD of 5 replicates.

An LPS-induced peritonitis mouse model was established to examine the anti-inflammatory effect of Ba-ME in vivo. Ba-ME (100 mg/kg) clearly decreased nitric oxide (NO) production (Figure 5a). Next, the mRNA expression levels and protein levels of AP-1 pathway-related factors were examined. Figure 5b indicates that the mRNA levels of COX-2 and CCL12 decreased. These observations indicate that Ba-ME has the capability to inhibit the progression of the AP-1 pathway, by proving its anti-inflammatory effect in vitro and in vivo.

## 3. Discussion

*Barringtonia augusta* has long been used as a folk medicine and is understood to act as an antioxidant [22]. However, a molecular mechanism that explains how Ba-ME inhibits inflammatory responses to the AP-1 signaling pathway has yet to be elucidated. We focused on how Ba-ME exerts its anti-inflammation function in vitro using LPS-stimulated RAW267.4 cells and an in vivo LPS-induced peritonitis mouse model.

The viability of HEK293T cells and RAW264.7 was examined to determine how Ba-ME produces anti-inflammatory effects without cytotoxicity (Figure 1) using an MTT assay [23]. LPS-stimulated TLR4 signaling modulates the COX-2 and pro-inflammatory cytokines by activating AP-1 pathways in macrophages [24,25]. Our goal was to determine whether Ba-ME mediates the downregulation of COX-2 and whether inflammatory cytokines are mediated in LPS-stimulated macrophages by suppressing AP-1 signaling.

Previously, O’Neill et al. demonstrated a method for identifying the essential components of TLR signaling that employed the transfection with a luciferase reporter gene construction and adaptor molecules in HEK cells [26]. As demonstrated in our studies [27,28], this method was obviously found to be reliable in investigating the functional activation of transcription factors. Following this approach, we transfected cells containing AP-1-Luc with adaptor molecules (TRIF and MyD88) to examine how Ba-ME suppresses AP-1 transcription activity (Figure 2). The AP-1-mediated luciferase activity was accelerated up to a factor of 6.5 by TRIF and MyD88 co-transfection while Ba-ME significantly and dose-dependently blocked this activity. Altogether, AP-1 activation is a pharmacologic target of the extract.

We exposed the RAW264.7 cells to LPS and measured the phosphorylated and total levels of c-Jun and c-Fos to comprehensively examine the effect on the AP-1 signaling pathway of Ba-ME. Figure 3a shows that Ba-ME can decrease levels of phosphorylated c-Jun and c-Fos in RAW264.7 cells under LPS stimulation conditions. It has been stated that MAPKs are able to control AP-1 activation, thus playing an important role in regulating LPS-induced inflammation [28,29]. In the same manner, our results suggest that Ba-ME specifically targets an upstream MAPK. We further analyzed the inhibitory effect of Ba-ME on MAPKs and their upstream signaling enzymes. We had to examine the effects of Ba-ME on the activity levels of the phosphorylated and total forms of p38, JNK, and ERK because there are many kinds of MAPKs (such as p38, JNK, and ERK) that activate AP-1 signaling pathways. Although the activity of p38 and ERK was not inhibited, Ba-ME inhibited the activity of kinase JNK at 5, 10, 15, 30, and 60 min, as shown in Figure 3c. The results indicate that Ba-ME targets were upstream signaling molecules in the AP-1 signaling pathways of anti-inflammatory activity. Brief experiments performed at 2, 3, and 5 min (Figure 4a) revealed that LPS enhanced the TAK1 phosphorylation, which takes place upstream of JNK. These results confirmed the MAPK inhibitory activity of Ba-ME.

To evaluate whether Ba-ME targets upstream AP-1 signaling molecules, we employed TAK1-overexpressing HEK293T cells. As shown in Figure 4b, Ba-ME suppressed the phosphorylation of TAK1. We examined whether TAK1 is the target of Ba-ME using CETSA experiments to identify interactions between Ba-ME and TAK1. The results confirmed that Ba-ME interacts with TAK1.

The LPS-induced peritonitis mouse model was employed for exploring the anti-inflammatory ability of Ba-ME in vivo. As shown in Figure 5, the Ba-ME treatment (100 mg/kg) improved LPS-induced peritonitis. The NO production assay (Figure 5a) helped us to confirm the suppressive effect of Ba-ME. Moreover, Ba-ME reduced inflammatory lesions, pro-inflammatory cytokines, and activation of AP-1 pathway-related proteins in the peritonitis model. These reports agree with the previously demonstrated effects of Ba-ME on mRNA production and active forms of AP-1 signaling molecules. Ba-ME was strongly peritonitis-protective in the mouse model of LPS-induced peritonitis. These results indicate that Ba-ME is a potential candidate for an anti-inflammatory medicine component.

## 4. Materials and Methods

### 4.1. Materials

*Barringtonia augusta* methanol extract (Lecythidaceae) was extracted from the leaf and stem of the plant from Vietnam. The phytochemical details of Ba-ME, including HPLC profile, are presented in the Supplementary Information. RAW264.7 and HEK293T cells were purchased from American Type Culture Collection (Rockville, MD, USA). Roswell Park Memorial Institute 1640 medium (RPMI 1640), Dulbecco’s modified Eagle’s medium, fetal bovine serum (FBS), and phosphate-buffered saline (PBS) were purchased from Capricorn Scientific GmbH (Ebsdorfergrund, Germany). TRIzol reagent was purchased from MRCgene (Cincinnati, OH, USA). MTT, sodium dodecyl sulfate, dimethyl sulfoxide (DMSO), polyethylenimine (PEI), and LPS (E. coli 0111: B4) were purchased from Sigma–Aldrich Co. (St. Louis, MO, USA). Penicillin–streptomycin and trypsin were purchased from HyClone (Logan, UT, USA). PCR premix and primers specific for COX-2, CCL12, CXCL3, CXCL9, and GAPDH were synthesized by Bioneer Inc. Antibodies specific for the phosphorylated and total forms of c-Fos, c-Jun, p38, ERK, JNK, TAK1, and β-actin were acquired from Cell Signaling Technology (Beverly, MA, USA).

### 4.2. Cell Cultures

A murine macrophage cell line (RAW264.7) was cultivated in RPMI 1640 medium supplemented with 10% heat-inactivated FBS and antibiotics (penicillin and streptomycin) at 37 °C in 5% CO_2_. Human embryonic kidney cell line (HEK293T) was cultured in cultured in DMEM medium with 5% heat-inactivated FBS and antibiotics (penicillin and streptomycin) at 37 °C in 5% CO_2_.

### 4.3. Mice

Male C57BL/6 Institute of Cancer Research mice (6 to 8 weeks old, 17 to 21 g) were obtained from Deahan Biolink (Chungbuk, Korea) and treated orally with Ba-ME (100 mg/kg) or ranitidine (40 mg/kg) twice per day for 3 days. Water and pellet chow were available ad libitum (Samyang, Daejeon, Korea). Studies (the permit number for experimentation on mice: SKKUIACUC2020-06-30-1) were performed following instructions established by the Sungkyunkwan University Institutional Animal Care and Use Committee.

### 4.4. Cell Viability Tests

The cytotoxicity of Ba-ME (5 × 10^5^ cells/mL) was assessed for 24 h, and HEK293T cells (2 × 10^5^/mL) were measured by MTT assays [6]. The cytotoxic effect of Ba-ME (25–50 μg/mL) was evaluated by a conventional MTT assay. The final concentration of MTT solution was 500 μg/mL. Cells were treated with Ba-ME for 24 h; 10 μL of MTT solution was added to cells 3 h prior to the end of the culture period. The addition of 15% sodium dodecyl sulfate to each well to dissolve the formazan stopped the assay. Absorbance at 570 nm was measured using a Synergy HT multi-mode microplate reader (BioTek Instruments, Inc., Winooski, VT, USA).

### 4.5. mRNA Analysis by Quantitative Reverse Transcription Polymerase Chain Reaction

RAW264.7 cells (1 × 10^6^ cells/mL) were treated with Ba-ME (100–150 µg/mL), and induction was executed with LPS (1 µg/mL) after 30 min. After 6 h of induction, RNA was extracted by a TRI reagent according to the manufacturer’s instructions and stored at −80 °C. A 1 µg sample of total RNA was used in a cDNA synthesis kit (Thermo Fisher Scientific, Waltham, MA, USA) following the manufacturer’s instructions [30,31]. The used primer sequences are listed in Table 1.

### 4.6. Plasmid Transfection and Luciferase Reporter Gene Activity Assays

HEK293T cells (2.5 × 10^5^/mL) were seeded in 24-well plates. The cells were transfected with plasmids encoding a luciferase gene (AP-1-Luc) with AP-1 promoter sites. MyD88 or TRIF genes were then co-transfected to further activate the luciferase genes. Transfections were performed using the polyethylenimine (PEI) method. After 24 h, we treated the transfected cells with Ba-ME (0–150 µg/mL). The harvested cells were lysed by freezing at −70 °C for at least 3 h. A luminometer was used to measure luciferase reporter activity [32].

### 4.7. Western Blot Analysis

RAW264.7 cells (1 × 10^6^/mL) were pretreated with Ba-ME (150 µg/mL) for 30 min, after which LPS induction (1 μg/mL) was processed for indicated times (2, 3, 5, 15, 30, and 60 min). HEK293T cells were overexpressed with TAK1 for 48 h and treated with Ba-ME (150 μg/mL) for 12 h. Western blot analyses, using whole lysates of the RAW264.7 and HEK293T cells, were performed using antibodies specific for each target protein. The primary antibodies (1:2500) to total and phosphoforms of c-Jun, c-Fos, JNK, p38, ERK, TAK1, and β-actin, and the secondary antibodies (1:3000) recognizing corresponding isotypes, were used, as reported previously [33,34]. The protein bands were detected with the help of an ECL system (Amersham, Little Chalfont, Buckinghamshire, UK).

### 4.8. Cellular Thermal Shift Assays

HEK293T cells were transfected by plasmids expressing TAK1 domain-deletion genes and treated with Ba-ME (150 μg/mL) or DMSO (as a control) for 24 h. After treatment, the cells were isolated and resuspended in PBS. The suspended cells were separated into seven PCR tubes with a volume of 100 µL and equal numbers of cells. Each PCR tube was heated for 3 min at a temperature gradient from 49 °C to 61 °C and then cooled to 25 °C for 3 min. We performed three rounds of freezing and thawing using liquid nitrogen and room-temperature water, as reported previously [35,36]. The samples were transferred into Eppendorf tubes and centrifuged at 12,000 rpm for 30 min. Protein samples were examined by Western blot analysis.

### 4.9. LPS-Induced Peritonitis Mouse Model

C57BL/6 male mice (*n* = 5 per group) were injected intraperitoneally with 1 mL of 4% thioglycollate broth for 4 days [37,38]. Ba-ME (100 mg/kg) suspended in 0.5% Na-CMC was administered orally to the thioglycollate-injected mice daily for 5 days using gavage. Acute peritonitis was induced in the thioglycollate-injected mice by intraperitoneal injection of 1 mL of LPS (10 mg/kg); peritoneal macrophages derived from the mice were collected and plated in RPMI 1640 medium at 1 day after LPS injection. The total RNA in peritoneal exudates was isolated with TRIzol reagent according to the manufacturer’s instructions and measured by an mRNA analysis by Quantitative Reverse Transcription Polymerase Chain Reaction.

### 4.10. Nitric Oxide (NO) Assay

Peritoneal macrophages were pre-treated with Ba-ME and then stimulated with LPS. The supernatant (100 µL) obtained was mixed with 100 µL of Griess reagent. The absorbance of this mixture was measured at 540 nm and a standard curve was employed to calculate the concentration of NO.

### 4.11. Isolation of Peritoneal Macrophage

After 5 days of oral administration to the thioglycollate-injected mice, we obtained the peritoneal macrophages by IP lavage. The isolated peritoneal macrophages (1 × 10^6^ cells/mL) were washed with RPMI1640 medium and they were cultured for 4 h at 37 °C in 5% CO_2_ in a humidified incubator.

### 4.12. Statistical Analysis

The data are presented as the mean and standard deviation of independent replicate experiments performed in triplicate for statistical comparisons. Statistical comparisons were examined by a Student’s *t*-test and a one-way analysis of variance. A *p*-value < 0.05 was considered statistically significant. All statistical analyses were performed using SPSS software.

## 5. Conclusions

**Figure 6:**
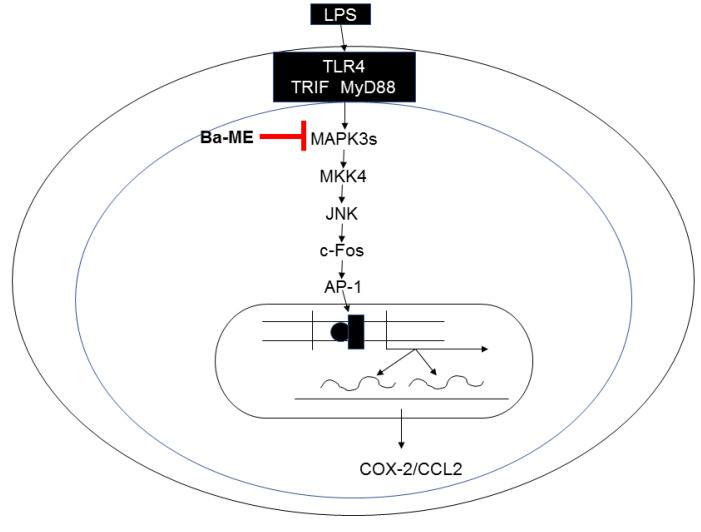
Signaling pathway scheme of Ba-ME’s anti-inflammatory action.

Through the above experiments, we proved that Ba-ME has an anti-inflammatory effect both in vitro and in vivo. Ba-ME effectively suppressed the expression of COX-2 and CCL12 in LPS-stimulated macrophages in a dose-dependent manner. Ba-ME inhibits the activation of AP-1 signaling, as shown by luciferase assay. Further analysis of kinase activities by in vitro assays and Western blotting confirmed that Ba-ME blocks the AP-1 pathway through the expression level of JNK. Employing an overexpression strategy, a CETSA showed that Ba-ME targeted TAK1 to inhibit macrophage-mediated inflammatory responses. In Figure 6, we summarize the inhibitory progression of the AP-1 pathway mechanism of Ba-ME to achieve the anti-inflammatory effects in vitro and in vivo. Our research suggests that Ba-ME could be a potential anti-inflammatory therapeutic.

## Figures and Tables

**Table 1:** Primer sequences for analysis of mRNA prepared from RAW264.7 cells used in RT-PCR.

Name	Direction	Sequence (5′ to 3′)
COX-2	Forward	CACTACATCCTGACCCACTT
	Reverse	ATGCTCCTGCTTGAGTATGT
CCL12	Forward	GCCTCCTGCTCATAGCTACC
	Reverse	CTTCCGGACGTGAATCTTCT
CXCL3	Forward	CCAACGGTGTCTGGATGTGT
	Reverse	TGGCCAGCCAAGGAATACTG
CXCL9	Forward	ACAGGTTGACTGATTGGCA
	Reverse	GCTSSSGGATTTGGCAGCTC
GAPDH	Forward	ACCACAGTGGATGCCATCAC
	Reverse	CCACCACCCTGTTGCTGTAG

## Data Availability

The data used to support the findings of this study are available from the corresponding author upon request.

## Supplementary Materials

The following are available online, Figure S1: HPLC profile of Ba-ME and standard flavonoid compounds (silibinin, genistein, and apigenin).

## Sample Availability

Sample of Ba-ME is available from the authors.
