# Supplementary material for: TAK1/AP-1-Targeted Anti-Inflammatory Effects of Barringtonia augusta Methanol Extract
Source: Molecules. 2021 May 20;26(10):3053. doi: 10.3390/molecules26103053 (PMC8160894; doi:10.3390/molecules26103053)
Supplement: Supplementary file 1 [file molecules-26-03053-s001.zip › molecules-1208212-supplementary.pdf]

Supplementary information

TAK1/AP-1-Targeted Anti-Inflammatory Effects of *Barringtonia augusta* Methanol Extract

Anh Thu Ha [1], Mi-Yeon Kim [2,*] and Jae Youl Cho [1,*]

1 Department of Integrative Biotechnology, and Biomedical Institute for Convergence at SKKU (BICS), Sungkyunkwan University, Suwon 16419, Korea; anhthu95.vn@gmail.com
2 School of Systems Biomedical Science, Soongsil University, Seoul 06978, Korea
* Correspondence: kimmy@ssu.ac.kr (M.-Y.K.); jaecho@skku.edu (J.Y.C.); Tel.: +82-2-820-0458 (M.-Y.K.);
+82-31-290-7868 (J.Y.C.)

Phytochemical details of Ba-ME

Methanol extracts (95%) of *B. angusta* (leaves and stems) was supplied by the Foreign Plant Extract Bank of the International Biological Material Research Center (Daejeon, Korea; http://ibmrc.re.kr). *B. angusta* (leaf and stems) was collected on October 2017 from Phu Quoc National Park, Phu Quoc district, Kien Giang province, Vietnam. Plant samples were collected and identified by Dr. Tran The Bach at the Institute of Ecology and Biological Resources (Hanoi, Vietnam). Voucher specimens, recorded as KRIB 0031699 and VK 3767, have been deposited at the herbarium of the Korea Research Institute of Bioscience and Biotechnology (Daejeon, Korea).

The phytochemical characteristics of Ba-ME were confirmed using HPLC analysis with silibinin, apigenin, and genistein as the standard compounds. The analysis used a system equipped with an HPLC (Jasco) and UV-Vis detector. Elution solvents were solvent A (2% acetic acid in H_2O and 0.1% formic acid in MeOH:H_2O = 10:90) and solvent B (0.5% acetic acid in H_2O:acetonitrile = 50:50 and 0.1% formic acid in MeOH:H_2O = 90:10). The gradient step of the solvent was from solvent A to solvent B, and a CAPCELL PAK C_{18} MG, 4.6 mm I.D. × 250 mm column was used

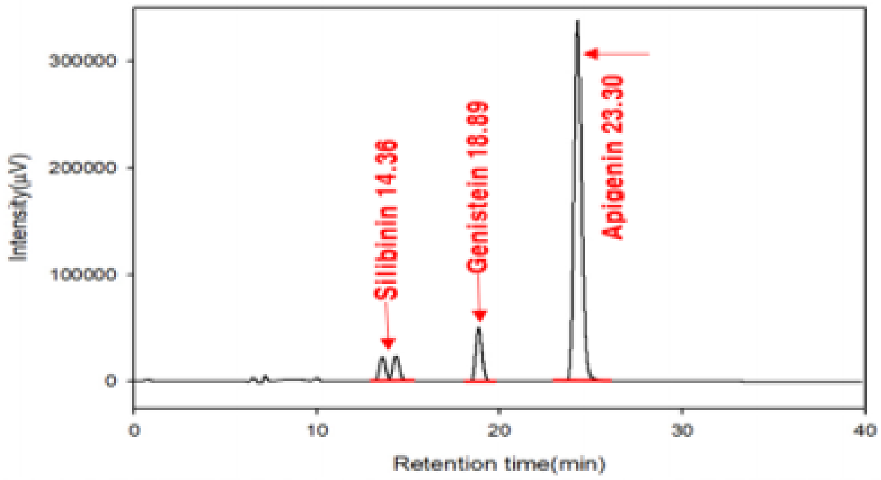

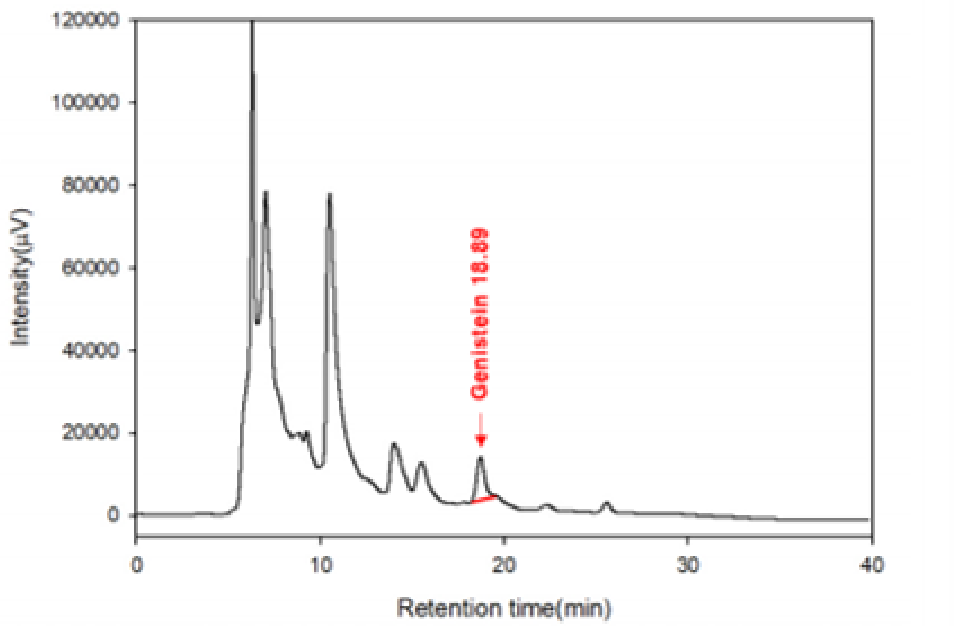

Supplementary Figure S1. HPLC profile of Ba-ME and standard flavonoid compounds (silibinin, genistein, and apigenin).
